# Two cases of debulking surgery for lower limb diffuse plexiform neurofibroma with transcatheter arterial embolisation

**DOI:** 10.1016/j.ijscr.2019.01.018

**Published:** 2019-01-29

**Authors:** Daiki Kitano, Takeo Osaki, Mika Nakasone, Tadashi Nomura, Kazunobu Hashikawa, Hiroto Terashi

**Affiliations:** Department of Plastic Surgery, Kobe University Graduate School of Medicine, Kobe, Hyogo, Japan

**Keywords:** Diffuse plexiform neurofibroma, Transcatheter arterial embolisation, Haemorrhage

## Abstract

**Introduction:**

Diffuse plexiform neurofibroma (DPN) in patients with neurofibromatosis type 1 (NF1) causes motility dysfunction in severe cases. Transcatheter arterial embolisation (TAE) is an effective haemorrhage control method in vascular tumour surgery.

**Presentation of case:**

We performed debulking surgery for DPN in the buttock and posterior thigh of two NF1 patients. Preoperative TAE with gelatine particles to tumour feeder vessels was conducted in both cases. Operative bleeding volumes were 500 and 4970 mL, respectively. In the latter case, the resection area extended to the upper poles of the buttocks, and the tumour invaded deeply into the surrounding tissues. Massive haemorrhage occurred, and internal iliac arterial balloon was inflated temporarily to further suppress the bleeding. Delayed wound healing due to TAE occurred; debridement and wound closure were required. Motor function improvement was confirmed in both patients.

**Discussion:**

Bleeding volumes varied because of highly developed collateral pathways and tumour invasiveness. As the upper pole of the buttock was perfused by the superior gluteal artery and its numerous collateral vessels, complete haemostasis was difficult despite adequate TAE. Because delineating the tumour border from the normal tissue was impossible due to the high tumour invasiveness, cutting into the hypervascular tumour was inevitable. As gelatine particles were absorbed but remained within the vessels, prolonged wound ischaemia and delayed healing occurred.

**Conclusion:**

Although TAE with gelatine particles and balloon occlusion were reliable haemorrhage control methods in debulking surgery for lower limb DPN, optimal haemorrhage control technique, compatible with haemostasis and wound healing, was desired.

## Highlights

• Debulking surgery was performed for DPN in two NF1 patients.
• Preoperative TAE with gelatin particles to tumour feeder vessels was conducted.
• TAE delayed wound healing; debridement and wound closure were required.
• Motor function improvement was confirmed in both patients.

## 1 Introduction

Type 1 neurofibromatosis (NF1) is an autosomal dominant genetic disorder characterised by various fibrous lesions in systemic organs [1]. Diffuse plexiform neurofibroma (DPN) is a cutaneous lesion of NF1 that poses cosmetic and functional problems [2]. Critical intratumoural bleeding secondary to trauma [3] and malignant transformation [4] have been reported in DPN patients. Some surgeons recommend complete resection in an early stage [5]. However, massive haemorrhage can occur during surgery due to vascularity and vulnerability of the tumour and surrounding tissues [6].

When large feeding vessels are evident, preoperative transcatheter arterial embolisation (TAE), aiming the reduction of the blood loss during the surgical procedure, is effective. This was firstly reported in 1983 [7]. In this study, we report two cases of DPN resection managed with preoperative TAE, and discuss the pros and cons of TAE in terms of haemostasis and wound healing.

This case report has been reported in line with the SCARE criteria [8].

## 2 Presentation of cases

**Fig. 1:**
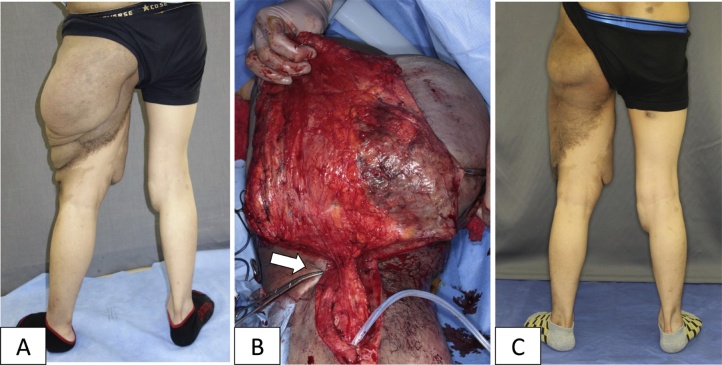
Case 1. A) Diffuse plexiform neurofibroma in the left buttock and thigh. B) The base of the pedunculated tumour was ligated to reduce the bleeding from the wound edge (white arrow). C) One year after debulking surgery. The diameter of the thigh was reduced by 30% approximately.

Case 1: A 20-year-old man with NF1 visited our clinic hoping to reduce the enlargement of his left lower limb. Examination confirmed a soft and drooping tumour encircling the thigh (Fig. 1A). DPN was diagnosed, and we planned a volume reduction surgery. To control haemorrhage, preoperative TAE to the superior gluteal artery and the deep femoral artery was performed with Serescue™ (Astellas Pharma Inc., Tokyo, Japan) by interventional radiologists.

Under general anaesthesia, debulking surgery was performed. The skin of the lateral thigh turned purple, probably because of ischaemia following TAE. Enclosing the discolouration area, a 3-kg tumour was resected above the fascia level. The tumour’s pedunculation and flexibility enabled us to temporarily ligate at the tumour’s base (Fig. 1B). The total amount of bleeding was 500 mL; transfusion was not needed. Postoperative course was satisfactory, and the patient was discharged 17 days after surgery. One year after the surgery, he attends the hospital on foot (Fig. 1C).

**Fig. 2:**
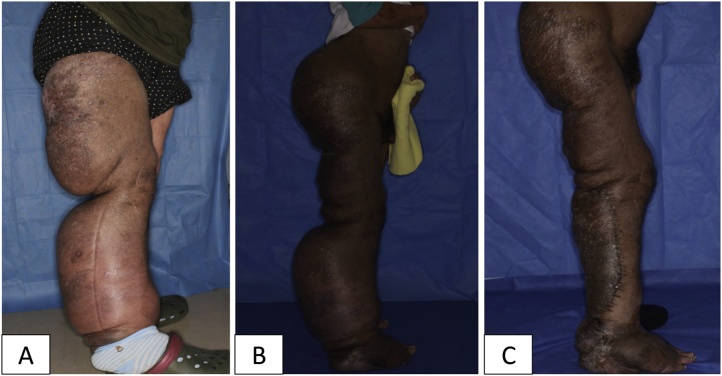
Case 2 (pre- and post-debulking surgery). A) Diffuse plexiform neurofibroma in the right lower limb. B) After the first surgery. The resection area was from the superior buttock to the posterior thigh. C) After the second surgery. With the tourniquet applied, the tumour below the knee was resected.

Case 2: A 40-year-old man, diagnosed with NF1 in his childhood, was referred to our department with complaints of walking difficulty. His right lower limb had begun to enlarge since his 30 s. Eventually, he had become unable to walk by himself because of his huge and heavy leg (Fig. 2A). We established that his symptom was caused by DPN in his leg, characterised by an elephant-like pigmented cutaneous tumour.

We planned serial excision with the resection area being from the buttock to the posterior thigh. Before the procedure, interventional radiologists performed TAE with Serescue™ to the feeding vessels originated from the superior and inferior gluteal arteries and the deep femoral artery of the affected side. An occlusion balloon was placed within the right internal iliac artery (IIA) to prevent uncontrollable critical haemorrhage.

**Fig. 3:**
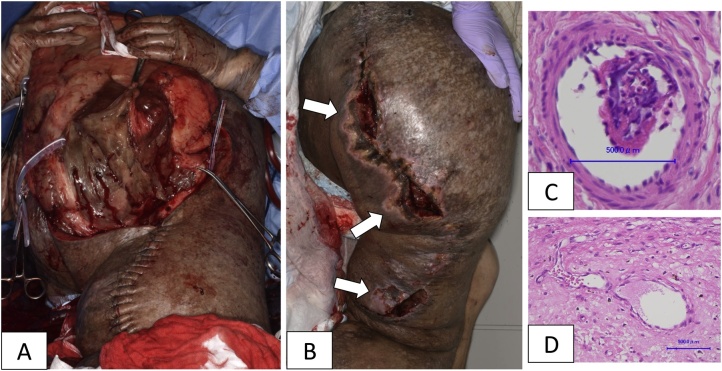
Case 2 (intraoperative and histological findings). A) The tumour had invaded deep into the normal tissue, which made it difficult to delineate the border of the tumour. B) Two weeks after the first surgery. The wound necrosis and dehiscence due to transcatheter arterial embolisation (white arrows). C) The occluded artery with gelatine particles in the resected specimen. D) The recanalized and stenosed feeder vessel in the tumour. This specimen was obtained during the debridement and wound closure 4 weeks after the embolisation.

Under general anaesthesia, we resected the tumour in the posterior thigh towards the head (Fig. 3A). In the buttock area, torrential bleeding from the wound occurred. The occlusion balloon in the IIA was inflated, and the bleeding was reduced temporarily. The total intraoperative blood loss was 4970 mL. We transfused 1960 mL of concentrated red cells and 1680 mL of fresh frozen plasma. The resected tumour weighed 5 kg with confirmed porous particles occluded in the arteries (Fig. 3C).

To prevent further bleeding from the wound edge, additional TAE was performed to the superior and inferior gluteal arteries immediately after surgery. The wound edge became gradually necrotic and dehiscent (Fig. 3B). Finally, we performed debridement and wound closure operation 4 weeks after the first surgery.

The second debulking surgery in the lower leg was conducted 4 months after the first debulking surgery. At this time, we could apply a tourniquet to the thigh area. The total blood loss during the second surgery was 420 mL, and TAE was not conducted. Wound healing was not delayed. The patient left the hospital walking by himself (Fig. 2C).

## 3 Discussion

DPN presents as a highly vascular tumour, and the number of feeder vessels increases with the proliferation of the tumour. Histological study has confirmed that the vascular walls become vulnerable because of thinning of the tunica media and fragmentation of the tunica elastic [9]. Hence, this tumour is prone to bleeding, and haemostasis is difficult. Especially in the lower limb DPN, massive haemorrhage is inevitable [10]. In our cases, the bleeding volumes were 500 and 4970 mL.

We assumed that the difference in the amount of bleeding between the two cases was caused by two reasons: location and invasiveness of the tumour. In case 2, the tumour resection area extended to the superior buttock. There is an abundant blood supply from the superior gluteal artery, a branch of IIA, in the superior buttock area [11]. The collateral pathway from the contralateral IIA via the iliolumbar artery has been reported [12]. We concluded that ipsilateral TAE and balloon occlusion are not enough to achieve haemostasis from the tumour in the buttock area, because of the nature of the numerous collateral pathways.

Second, the invasiveness of the tumour was markedly different in the two cases. The tumour invaded into the muscle and its surrounding soft tissues to a higher degree in case 2 than in case 1. As delineating the tumour margin in deeply infiltrative DPN is almost impossible, unintentional intralesional resection is not rare [13]. Therefore, we inferred that one of the reasons of high-volume blood loss in case 2 was cutting into the well-vascularised tumour.

The efficacy of TAE for haemorrhage control in surgical resection of lower limb DPN has been reported in several studies [7,14]. Among various materials used for TAE, gelatine particles made from skin and ligament of other animals were introduced as absorbable haemostatic agents in 1946 [15]. In this study, we adopted Serescue™, porous gelatine particles available in Japan. The reported mean diameter of occluded arteries with gelatine particles was 0.5 mm [16]. Also, we confirmed the gelatine particles within the 0.5-mm-sized arteries of the resected tumour (Fig. 3C).

Although gelatine particles are reliable haemostatic materials, focal ischaemia and necrosis of normal tissues are expected side effects of embolisation [17]. Gelatine particles in occluded arteries are thought to be removed by phagocytosis. Local tissues are found to be ischaemic for 2 weeks until vessel recanalisation was achieved. In addition, the recanalised vessel lumens are stenosed as a result of transluminal inflammation and smooth muscle migration due to foreign body reaction [18]. Delayed wound healing in case 2 can be attributed to TAE with gelatine particles. In fact, the stenosis of recanalised feeder vessels in the specimens of debridement was microscopically confirmed (Fig. 3D).

We struggled to achieve both adequate haemostasis and wound healing. As mentioned above, IIA receives blood supply from the contralateral side. Therefore, effective haemostasis can be expected in the buttock area by the intervention of bilateral IIAs. However, TAE with gelatine particles to bilateral IIAs leads to extensive necrosis of skin and muscle tissue in the buttock [19]. This can be explained by the infarction induced by gelatine particles remaining within the vessels after a certain period of time. On the contrary, balloon occlusion does not cause prolonged ischaemia and delay in wound healing postoperatively, because the balloon can be deflated as needed. In case 2, we placed the occlusion balloon to the unilateral IIA. In the field of obstetrics and gynaecology, balloon occlusion of bilateral IIAs has been reported to be an alternative haemorrhage-control method [20]. Further studies about indications and effects of balloon occlusion of bilateral IIAs in the debulking surgery of lower limb DPN are desired.

## 4 Conclusions

We adopted TAE with gelatine particles and unilateral balloon occlusion as haemorrhage control for the debulking surgery of lower limb DPN. Development of the ideal method for reducing blood loss to ensure the balance of haemostasis and wound healing is necessary.

## Conflicts of interest

The authors have no conflicts of interest to declare.

## Funding

None.

## Ethical approval

This study was exempt from ethical approval in Kobe University Hospital.

## Consent

Written informed consent was obtained from the patients for publication of this article including all figures. A copy of the written consent is available for review by the Editor-in-Chief of this journal on request.

## Author contribution

Daiki Kitano, Takeo Osaki, Mika Nakasone and Tadashi Nomura performed the operations. Daiki Kitano and Takeo Osaki drafted the manuscript. Tadashi Nomura, Kazunobu Hashikawa and Hiroto Terashi supervised the operations and this case report.

## Registration of research studies

Research Registry.

UIN: researchregistry4516.

## Guarantor

Takeo Osaki.

## Provenance and peer review

Not commissioned, externally peer-reviewed.
